# The Effectiveness of an Acceptance and Commitment Therapy and Mindfulness Group Intervention for Enhancing the Psychological and Physical Well-Being of Adults with Overweight or Obesity Seeking Treatment: The Mind&Life Randomized Control Trial Study Protocol

**DOI:** 10.3390/ijerph18094396

**Published:** 2021-04-21

**Authors:** Idoia Iturbe, Eva Pereda-Pereda, Enrique Echeburúa, Edurne Maiz

**Affiliations:** Department of Clinical and Health Psychology and Research Methodology, Faculty of Psychology, University of the Basque Country (UPV/EHU), 70 Tolosa Avenue, 20018 Donostia-San Sebastián, Spain; idoia.iturbe@ehu.eus (I.I.); eva.pereda@ehu.eus (E.P.-P.); enrique.echeburua@ehu.eus (E.E.)

**Keywords:** acceptance and commitment therapy, mindfulness, obesity, overweight, well-being

## Abstract

Although several interventions that target obesity have been examined, the success of these interventions in generating and maintaining positive results has yet to be confirmed. This study protocol therefore presents a trial aimed at analyzing the effectiveness of a well-being-centered acceptance and commitment therapy (ACT)- and mindfulness-based group intervention following the valued-based healthy living (VHL) approach (Mind&Life intervention) for individuals experiencing overweight-related distress. A randomized controlled trial with two parallel groups will be conducted in 110 adults attending primary care units with overweight or obesity. Participants will be randomly allocated to one of the two study conditions. Interventions will either be the treatment as usual (TAU) or the Mind&Life intervention—an ACT- and mindfulness-based intervention—plus the TAU intervention. Quality of life, weight self-stigma, general health status, eating habits, physical activity, eating behavior, anthropometric, body composition, cardiovascular, and physiological variables, as well as process variables, will be examined at baseline, posttreatment, 6-month follow-up, and 1-year follow-up. This trial aims to offer a novel psychological approach for addressing the psychological and physical impairments suffered by people with overweight or obesity in the current environment. ClinicalTrials.gov identifier: NCT03718728.

## 1. Introduction

Obesity is a chronic disease of multifactorial etiology, and due to a progressive increase in prevalence in recent decades, this condition is now regarded as a worldwide public health issue [1]. The excessive accumulation of body fat is clearly harmful to health, as it is linked to numerous noncommunicable diseases including diabetes and cardiovascular complications [2]. Furthermore, the economic costs resulting from obesity are estimated to represent around 0.7–2.8% of a country’s total health care expenditure [3].

This health condition has a considerable impact on psychological well-being, as individuals categorized as obese often experience multiple forms of discrimination, which adversely affects subjective well-being and health-related quality of life [4,5]. Moreover, weight-related stigma and weight self-stigma are linked to psychological distress (i.e., depression and anxiety) [6], while internalized stigma is also associated with a lower quality of life [7].

Primary care units in the Spanish health care system currently use standard weight-related interventions based on diet and physical activity guidelines. Although these interventions appear to be effective for initial weight loss, long-term weight management is a challenge due to weight regain, with multiple factors determining weight maintenance [8,9]. These interventions, however, have proven to be successful in reducing weight regain for up to 12 months when compared with controls [10]. Additionally, the long-term effectiveness of lifestyle interventions for improving emotional aspects and quality of life is questionable [11,12]. One possible explanation for this could be that health-related quality of life is positively associated with the amount of weight lost, and as previously mentioned, these interventions are ineffective for weight loss maintenance [13].

Several studies have shown that psychological factors play a determining role in weight management behaviors [14]. Whereas interventions based on various psychological approaches have been evaluated in terms of effectiveness for weight loss [15], the results have not led to consistent conclusions. For instance, cognitive-behavioral therapy (CBT) has been considered the most preferred intervention for obesity as it has been shown to be effective in improving cognitive and behavioral outcomes, along with weight loss [16,17,18]. In contrast, studies have also shown the ineffectiveness of CBT in targeting emotional aspects [17,19] and weight loss in both the short and long term [19,20]. Motivational interviewing (MI) has also yielded mixed results, with some studies demonstrating a greater efficacy for weight management compared with a control group and others indicating nil to modest efficacy [21,22,23]. Moreover, although the results suggest that MI promotes overall quality of life in people with overweight or obesity, this appears to depend on weight loss [24].

Third-wave cognitive-behavioral therapies in weight management treatments have shown more promise than standard behavioral interventions in the short, medium, and long term [25]. One such therapy is acceptance and commitment therapy (ACT), which is a relatively new psychological approach based on functional contextualism [26]. Within the context of weight management, ACT proposes the alignment of healthy behaviors with deeply held personal values and commitment to them, which function as an anchor when facing challenging situations and promote motivation to persist behaving healthfully [27,28,29]. Likewise, ACT suggests mindfulness to facilitate awareness of unwanted internal or external events that could trigger unhealthy behaviors (e.g., emotional hunger, self-stigmatizing thoughts) and acceptance to address those cues rather than trying to avoid, control, or alter them [27,28,29].

The use of ACT has shown the most consistent evidence of effectiveness for weight management in adults with overweight or obesity after 18 months [25]. One ACT-based study reported that those who received acceptance-based strategies showed an increased probability of maintaining a 10% weight loss and experienced a higher quality of life at 2-year follow-up [30]. Acceptance-based strategies also appear to be particularly beneficial for individuals who show high levels of emotional eating [31,32], a psychological factor that has a direct negative impact on weight management [33]. Furthermore, Sairanen et al. suggested that ACT-based interventions increase weight-related psychological flexibility, intuitive eating, and mindfulness [34]. Interventions based on the latter have also been effective for reducing weight and targeting psychological outcomes such as eating behaviors, problematic eating attitudes, depression, and anxiety [35,36]. In addition, developing mindfulness abilities protects an individual from transforming weight-related stigma into unpleasant emotional symptoms [37].

According to the existing literature, studies evaluating the effect of ACT and/or mindfulness in people with overweight or obesity have shown that these strategies are not only effective for quality of life enhancement [25,38] but are also the most successful for targeting emotional eating [33]. Moreover, weight-related experiential avoidance—a process directly targeted in ACT interventions and mindfulness—mediates changes in emotional eating and weight control [39]. Further, these positive results appear to be independent of weight-related outcomes [30]. Besides, ACT shows promise for reducing weight self-stigma [40,41,42] and psychological distress [43,44], although some inconsistent findings have also been found in the latter variable [45].

Nowadays, two distinct but related ACT approaches focusing weight management issues are distinguished [29]: Acceptance-based behavioral treatment (ABT) and values-based healthy living (VHL). On the one hand, ABT is built upon standard behavioral weight loss interventions, focusing on the usual weight-control goals. However, this approach considers the inclusion of acceptance-based strategies and clarification and commitment to values to sustain weight control efforts. On the other hand, VHL purposes to sweep away the usual weight loss agenda and is centered mainly on clients’ overall quality of life targeting broader values work. That is, this perspective’s aim is to place healthy behaviors firmly into meaningful living context rather than focusing on a concrete weight loss.

In this line, more recently, the well-established weight-normative approach for addressing obesity-related issues has been brought into question. This perspective places weight as the principal determinant of health or disease. Therefore, efforts to enhance physical well-being are focused mainly on weight loss. However, the weight-normative approach involves an increase in perceived and internalized weight stigma, which in turn decreases psychological well-being and physical health while promoting unhealthy eating behaviors [46]. Accordingly, two ACT-based studies in line with the VHL perspective adopting psychological well-being-related variables as primary outcomes found positive results at posttreatment and 3-month follow-up in psychological distress, quality of life, weight-related stigma, self-criticism, eating behaviors, and physical exercise [43,44]. Moreover, a secondary outcome of these therapies was that they were effective in reducing weight, which suggests that these programs could represent another useful strategy for promoting psychological and physical well-being. However, more studies of interventions based on the VHL perspective with longer follow-ups are needed to assess their efficacy in generating and maintaining positive results for obesity-related issues.

The primary aim of this study is to evaluate the effectiveness of a well-being centered ACT- and mindfulness-based group intervention following the VHL perspective in conjunction with the treatment as usual (TAU) (Mind&Life intervention) in comparison with TAU alone. The measured outcomes will be quality of life, weight self-stigma, general health, eating habits, physical activity, and eating behaviors (i.e., emotional eating, restrictive eating, and external eating) of people with overweight or obesity in the short, medium, and long term. It is hypothesized that, in comparison with people receiving TAU (control group), those receiving the Mind&Life intervention (experimental group) will show a greater improvement in these outcomes in the short, medium, and long term.

A secondary goal of this study is to examine the effectiveness of the Mind&Life intervention when considering anthropometric measures (i.e., weight, body mass index (BMI)), body composition measures (i.e., body fat percentage, waist and hip circumference), cardiovascular measures (i.e., blood pressure), and physiological measures (i.e., lipid and glucose profile) at the short, medium, and long term. It is anticipated that the experimental group will show a greater improvement in these variables than the comparison group in the short, medium, and long term. In addition, this trial aims to analyze the mediating and moderating role of various process measures (i.e., psychological flexibility, weight-related psychological flexibility, mindfulness abilities, self-compassion, valued living, and cognitive fusion) in the relationship between the Mind&Life intervention and all outcome measures. We will also examine the mediating and moderating role of weight self-stigma and emotional eating in the relationship between the Mind&Life intervention and all outcome measures.

## 2. Materials and Methods

This protocol was developed basing on the Standard Protocol Items: Recommendations for Interventional Trials (SPIRIT) statement [47] and registered at ClinicalTrials.gov with reference number: NCT03718728.

### 2.1. Trial Design and Setting

The current trial was designed as a randomized, controlled, nonblinded superiority trial with 2 parallel groups. Randomization will be blocked with a 1:1 allocation. The study will be located in the Faculty of Psychology of the University of the Basque Country (UPV/EHU; Donostia-San Sebastián, Spain).

### 2.2. Patient Involvement

This study was designed by the Mind&Life team, a multidisciplinary group based at the University of the Basque Country (UPV/EHU). This group is composed of researchers, psychologists, and nutritionists working in partnership with nurses and doctors from the Basque Health Service, along with physical activity and sport specialists from the Donostia-San Sebastian council representing the various interests of patients and caregivers.

### 2.3. Participants

#### 2.3.1. Recruitment

The research group will contact the doctors and nursing staff from primary care units in Donostia-San Sebastian with the objective of actively involving them in the recruitment process. Information sheets will be provided which will be distributed among possible participants attending primary care units. Those interested in participating in the study will then contact a research team member, who will then confirm the eligibility of the participant.

#### 2.3.2. Sample

##### Eligibility Criteria

Participants will be eligible if they (1) have overweight or obesity (estimated by BMI ≥ 25 kg/m^2^ and classified by the International Obesity Task Force, IOTF); (2) are between 20 and 70 years old; and (3) seek weight management treatment. Participants will be excluded if they (1) have an eating disorder diagnosis such as binge eating disorder, anorexia, or bulimia; (2) have any psychiatric disorder or intellectual disability that prevents them from taking part in the intervention; or (3) have insufficient knowledge of the language in which the intervention will be implemented.

##### Sample Size

Sample size and power estimation were calculated for 5% alpha error, a 2-tailed test, and 80% statistical power (beta error = 20%). The trial uses a comparison group and experimental group design for which pretreatment–posttreatment differences are expected to have an effect size of 0.68 (Cohen’s *d*) for quality-of-life index. The latter has been elected due to the VHL approach of Mind&Life intervention, since among all primary outcomes, quality-of-life index could best reflect values’ work impact. This effect size is based on that of a previous trial studying an acceptance, mindfulness, and compassion-based group intervention program for people struggling with weight-related issues [44]. Since dropout rates of 4–20% have previously been observed, in the present trial, the sample size and statistical power calculations are based on a 20% attrition rate. Hence, it is estimated that 55 participants per group (110 in total) will be needed to evaluate one of the main outcomes of the study, that is, quality of life.

### 2.4. Interventions

Eligible participants will be randomly allocated to either the Mind&Life or the TAU intervention, both of which are of 5 months duration.

#### 2.4.1. Treatment as Usual

This intervention consists of 5 monthly individual sessions of 30 min and includes nutritional advice and recommendations for physical activity. The sessions will be delivered by a nutritionist and will take place in a laboratory of the Faculty of Psychology of UPV/EHU. All sessions will follow the same structure. First, the participant’s anthropometric measures will be taken, after which their physical state and progress during the last month will be examined. The dietetic and physical activity registration form will then be reviewed. Finally, the nutritionist will provide information about a specific topic. An overview of the content of each session is displayed in Table 1. Participants will be provided with a folder with their initial anthropometric assessment report and session materials, as well as a card and a pen to note down the measures taken at each session. They will also be asked to keep a record of daily dietary habits and physical activity, which will be reviewed in each session.

#### 2.4.2. Mind&Life Intervention

This group intervention is a combination of ACT- and mindfulness-based intervention following a VHL approach centering primarily on valued living. It consists of 15 two-hour sessions, the first 10 being held weekly and the last 5 being held biweekly. At the end of the treatment, the participants will be followed up with a monthly telephone call for a period of 6 months in order to review personal values, value-driven behaviors, and the integration of mindfulness into their everyday life [48]. The intervention has been developed by consulting ACT manuals [49,50], including those that specifically target weight loss [51,52], an ACT metaphor and exercises book [53], along with a mindfulness [54] and mindful eating [55] exercise book. Moreover, Kg-Free [44] has been taken as reference for the elaboration of Mind&Life intervention, although there are significant differences in participants’ inclusion criteria (e.g., age and gender), follow-up length, and intervention components (e.g., compassion’s presence, values revising frequency or mindfulness practice). Two psychologists with training in third-wave therapies will be responsible for conducting the sessions in small groups (from 12 to 14 participants), which will take place in a classroom of the Faculty of Psychology of UPV/EHU where chairs will be arranged in a circle. The Mind&Life intervention is characterized by avoiding overemphasis on weight loss during the whole intervention process. Instead, the intervention targets psychological well-being by clarifying personal values and fostering value-driven behaviors. Whereas reflecting on the personal values that underpin self-care will be of great importance, the interventionists will take care to prevent the participants from basing their personal development on weight-related aims. All sessions will follow a common structure: Mindfulness practice (general or mindful eating), an explanation of the session content with the use of metaphors or experiential exercises, and an explanation of homework tasks. The specific components of each session are presented in Table 2. Participants will be supplied with a manual containing a summary of each session, homework instructions, and contact information. Further, at the end of each session, the interventionists will send participants an audio file with the mindfulness exercise in order to facilitate daily practice. Additionally, they will be asked to record their daily mindfulness practice in either paper or online format.

#### 2.4.3. Concomitant Interventions

This trial will be incompatible with receiving any kind of additional psychological or dietetic treatment. Nevertheless, drugs for controlling blood pressure, diabetes, or cholesterol will be permitted.

### 2.5. Randomization

Participants will be randomly assigned to either the experimental or the comparison group using a 1:1 allocation ratio. Blocked randomization will be used, and a computerized random sequence generator (www.random.org) will determine the sequence of each block. First, the size of the block will be established, after which a member of the research team not involved in the assessment sessions or the delivery of the treatment will write down the allocation sequence of each block on a sheet of paper which will be concealed in an opaque, sealed envelope. At the time of allocation, once participants have provided consent, the research team member in charge of the assessments and delivery of the treatment will open the corresponding envelope to reveal the allocation sequence to assign each participant to the corresponding group.

### 2.6. Plan to Promote Participant Adherence or Retention

Given the importance of adherence to treatment and retention in the study, the following strategies will be implemented. In the first assessment session, together with providing the information sheet and informed consent form, researchers will highlight the importance of adhering to the treatment, which will also be mentioned in the first treatment session. In particular, the researchers will emphasize the benefit of attending sessions, doing homework, and practicing mindfulness, as well as recording their daily physical activity, diet, and mindfulness practice in their everyday life. Further, in order to promote adherence to treatment, the interventionists will send a message to all participants to remind them of each TAU appointment and group session with Mind&Life once they are held biweekly. Similarly, in order to favor participant retention, in each dietetic assessment session, all participants will be given oral (and written, if requested) feedback explaining their physical state and overall development (based on anthropometric indicators). Additionally, it is expected that both the interventionists and researchers will be available to be contacted by telephone or email at any time throughout the entire study period. In all cases, attendance and attrition will be recorded with the aim of eventually comparing results based on these rates.

### 2.7. Masking

Only the person responsible for collecting anthropometric, cardiovascular and dietetic data will be blinded, since knowledge of the treatment being given to participants could influence the measurement of outcomes. In contrast, the person responsible for the measurement of the other variables will not be blinded due to the fact that self-report instruments will be employed. Similarly, blinding of the psychologists and participants will not be possible. In the case of the participants, from the moment they read the informed consent document, they will inevitably be aware of the existence of the 2 interventions.

### 2.8. Data Collection and Assessment Plans

**Figure 1:**
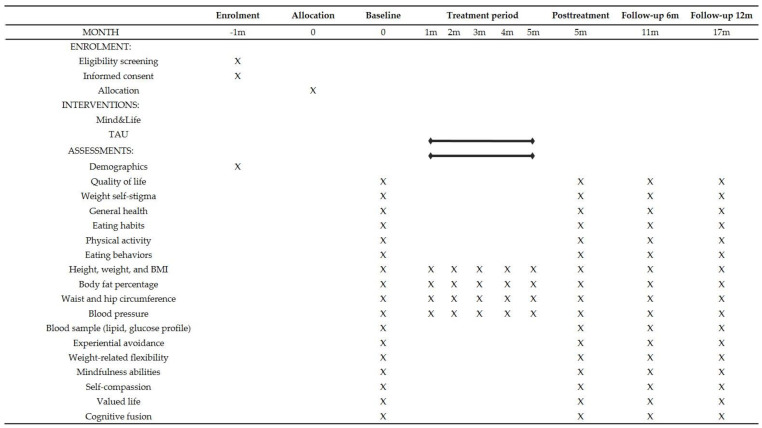
SPIRIT flow diagram: Schedule of events for the Mind&Life study.

For the present trial, the outcomes will be measured at baseline, posttreatment, 6-month follow-up, and 1-year follow-up. Weight, body fat percentage, waist and hip circumference, and blood pressure will also be measured monthly during the intervention period (see Figure 1 for details of the assessment schedule). Self-reported measures will be collected using the *Encuesta Fácil* online platform (www.encuestafacil.com). Assessment sessions will take place in a computer room and a laboratory in the Faculty of Psychology at UPV/EHU, located in Donostia-San Sebastian. Blood samples will be collected by a nurse in each participant’s primary care unit. A nutritionist will be responsible for evaluating anthropometric and dietetic aspects, having been trained specifically for that task. A psychologist will be in charge of conducting the assessment sessions for the other variables evaluated in the study, having also been trained for that purpose. The evaluated outcomes and the properties of the assessment instruments to be used are detailed below.

#### 2.8.1. Enrollment Criteria and Baseline Characteristics

##### Demographics

Data related to the participants’ gender and age will be collected.

##### Weight-Related Information

Information will be requested about the participants’ self-reported weight and height (in order to calculate self-reported BMI and exclude those that clearly do not meet the inclusion criteria), weight history, and the existence of any excess weight-related illnesses.

##### Participation Motivation

People interested in participating in the study will be asked about their reasons for taking part, with the purpose of establishing their motivation for doing so.

#### 2.8.2. Primary Outcomes and Measures

##### Quality of Life

Quality of life will be assessed by the Spanish version of the Impact of Weight on Quality of Life-Lite Questionnaire (IWQOL-Lite) [56], an instrument that measures quality of life in people with obesity. This 31-item questionnaire comprises 5 subscales that measure weight-related concerns across 5 domains: Physical function, self-esteem, sexual life, public distress, and work. The whole scale and the 5 subscales have shown good reliability (Cronbach’s *α* = 0.88–0.95).

##### Weight Self-Stigma

Weight self-stigma will be assessed by the Spanish adaptation of the Weight Self-Stigma Questionnaire (WSSQ) [57], which is a 12-item questionnaire used to assess weight-related self-stigma in people with overweight or obesity, and, in addition to the total score, comprises the following two subscales: self-devaluation and fear of enacted stigma. The whole scale and the subscales show good internal consistency (Cronbach’s *α* = 0.81–0.88).

##### General Health

Current general health status will be assessed using the Spanish version of the General Health Questionnaire-28 (GHQ-28) [58]. In addition to the total score, this measure consists of 28 items comprising the following 4 subscales: Somatic symptoms, anxiety/insomnia, social dysfunction, and severe depression. Both the total score and subscales have shown good internal consistency (Cronbach’s *α* = 0.91–0.97) [59].

##### Eating Habits

Eating habits will be assessed by two 24-h recalls (on alternate days) and the 14-item Mediterranean Diet Assessment Tool [60], a 14-item questionnaire that evaluates adherence to the Mediterranean diet. Eating habits will be examined through the Easydiet© software package (online version 2019) developed by the Spanish Centre for Higher Studies in Nutrition and Dietetics (CESNID).

##### Physical Exercise

Physical exercise will be estimated by the Spanish short version of the International Physical Activity Questionnaire (IPAQ) [61], which is a questionnaire comprising 7 items that assess physical activity levels. This questionnaire provides information about the time an individual has spent walking, doing moderate intensity exercise, vigorous intensity exercise, and sedentary activities in the last 7 days. The short Spanish version of the questionnaire has shown very good reliability (Spearman’s *ρ* = 0.81) among the US population.

##### Eating Behavior

Eating behavior will be assessed by the Spanish version of the Dutch Eating Behavior Questionnaire (DEBQ) [62]. This comprises 33 items and 3 subscales related to various intake behaviors: Emotional, external, and restrained eating. All of its subscales have shown good internal consistency (Cronbach’s *α* = 0.84–0.94).

#### 2.8.3. Secondary Outcomes and Measures

##### BMI

Weight will be measured using a bioelectrical impedance analysis technique with a Tanita body composition monitor (Tanita Corp., Tokyo, Japan), and height will be measured using a wall-mounted stadiometer (SECA 220, Hamburg, Germany). These values will then be used to calculate BMI, and the participants will be classified as having overweight or obesity according to the IOTF cutoff points.

##### Body Fat Percentage

Body fat percentage will be estimated using a bioelectrical impedance analysis technique with a Tanita body composition monitor (Tanita Corp., Tokyo, Japan).

##### Waist and Hip Circumference

Waist and hip circumference will be measured by inelastic tape (Holtain^®^, Crymych, UK).

##### Blood Pressure

Blood pressure will be measured with a sphygmomanometer (Geratherm^®^, Geschwenda, Germany).

##### Lipid Profile

Total, LDL-, and HDL-cholesterol will be assessed with a blood test.

##### Glucose Level

Glucose levels will be measured by spectrophotometry.

##### Insulin Sensitivity

Insulin sensitivity will be measured using homeostasis model assessment (HOMA).

#### 2.8.4. Process Outcomes and Measures

##### Experiential Avoidance

Experiential avoidance will be assessed by the Spanish version of the Acceptance and Action Questionnaire-II (AAQ-II) [63], which is a 7-item questionnaire that evaluates general experiential avoidance and psychological inflexibility. This instrument has shown good internal consistency (Cronbach’s *α* = 0.88).

##### Weight-Related Flexibility

Flexibility in relation to difficult weight-related thoughts and feelings will be assessed by the Spanish adaptation of the Acceptance and Action Questionnaire for Weight-Related Problems (AAQ-W) [64]. This is a 22-item questionnaire that evaluates the acceptance of weight-related feelings, defusion from weight-related thoughts, and the degree to which thoughts and feelings interfere with valued action. The original version of the AAQ-W has shown good internal consistency (Cronbach’s *α* = 0.86).

##### Mindfulness Abilities

Mindfulness abilities will be assessed by the Spanish version of the Five Facets Mindfulness Questionnaire (FFMQ) [65], which is a 39-item questionnaire that assesses the capacity of an individual to engage in mindfulness practice. In addition to a total score, it comprises 5 subscales referring to 5 facets of mindfulness: Observing, describing, acting with awareness, nonjudging, and nonreactivity. The FFMQ total score and the 5 subscales have shown good internal consistency (Cronbach’s *α* = 0.80–0.91).

##### Self-Compassion

Self-compassion will be assessed by the Spanish short version of the Self-Compassion Scale (SCS) [66], which is a 12-item questionnaire that evaluates the way in which an individual shows kindness and understanding toward oneself in moments of turmoil. In addition to the total score, it comprises 6 subscales that assess components of self-compassion across 3 related facets: Self-kindness/self-judgment, common humanity/isolation, and mindfulness/overidentification. The whole scale and the 6 subscales have shown adequate internal consistency (Cronbach’s *α* = 0.71–0.85).

##### Valued Life

The fit between an individual’s actual activities and their valued behavioral pattern will be measured by the Spanish adaptation of the Valued Living Questionnaire (VLQ) [67], which is a 2-part instrument used to assess valued living. In the first part, subjects rate the importance of 10 domains of living while the second part rates how consistently the individual has lived in accord with the valued behavioral pattern within each domain over the past week. The instrument has shown adequate test-retest reliability [68].

##### Cognitive Fusion

Cognitive fusion will be measured by the Spanish version of the Cognitive Fusion Questionnaire (CFQ) [69], which is a 7-item questionnaire that evaluates the extent to which an individual is psychologically entangled with and dominated by the form or content of his/her thoughts. The scale has shown good internal reliability (Cronbach’s *α* = 0.87).

### 2.9. Data Management

Upon enrollment in the study, demographic information will be entered electronically into an Excel file to which only 2 investigators will have access. Likewise, data collected at baseline, posttreatment, 6-month follow-up, and 1-year follow-up will be accessible to only 1 investigator. Informed consent sheets, questionnaires administered on paper format, blood test results, and data related to anthropometric indicators, blood pressure, and 24-h recalls will be kept in locked cabinets which can only be accessed by the same researcher, although the nutritionist will also be aware of the latter piece of information. The information stored online will be copied onto the hard disk of the project. To ensure confidentiality, pseudonymization will be used, where participants will be provided with an ID number after signing the informed consent form, which will be used for assessments. Data forms and identification information will be stored separately, and once all data have been collected, identification information will be eliminated. Participant data will be stored in a file that will be the responsibility of the University of the Basque Country (UPV/EHU). Participants will be provided with information required to access their personal data utilized in the study, and they will be informed of their rights. Data will be preserved unless the interested individual requests its suppression.

### 2.10. Data Analysis

Primary, secondary and process-outcomes will be analyzed using the statistical program SPSS version 26.0 for Windows (IBM Corp., Armonk, NY, USA). Taking into account the aims of the study, the effects of the Mind&Life program will be examined by analysis of variance (ANOVA) and/or covariance analysis (ANCOVA), with group as the independent or fixed variable (experimental or control) and all study outcomes as dependent variables. If any difference in relation to a variable exists at baseline, it will be introduced as a covariate in the analyses. Likewise, when making comparisons at follow-up points (6-month and 1-year follow-up), pretreatment–posttreatment changes will also be introduced in the analyses as covariates. To estimate effect sizes, Cohen’s *d* will be calculated where values between 0.2 and 0.4 will indicate small effects; between 0.5 and 0.7, medium effects; and above 0.8, large effects [70]. Additionally, we will use multiple regression in order to predict the variables that best explain changes in the quality-of-life criterion variable. For mediation and moderation analyses, AMOS software will be used. For each test, values of *p* ≤ 0.05 will be considered significant.

### 2.11. Dissemination

The results of the present trial will be disseminated via journal publication and by talks and posters presented in international meetings related to obesity, third-wave behavioral therapies, or health psychology. The results of the trial will be disseminated regardless of the magnitude or direction of the outcomes. All investigators that participate actively in the study will have the opportunity to collaborate on published articles, even though final authorship will be determined by their contribution to the work.

## 3. Discussion

In this paper, we presented a study protocol of an ACT- and mindfulness-based group intervention with VHL perspective that aims to enhance the psychological and physical well-being of individuals with overweight or obesity seeking treatment. Providing an effective treatment for obesity-related issues is a matter of urgency due to the growing prevalence of this condition, along with the associated psychological suffering, health complications, and resulting economic costs [71]. Nevertheless, to date, inconclusive results have been obtained regarding the effectiveness of various lifestyle or psychological approaches, particularly in the long term [8,20]. ACT proposes a rather different approach to understanding the underlying causes of psychological distress and any kind of maladaptive behavior, which are closely linked to cognitive fusion and experiential avoidance [26]. Therefore, to address the tendency to respond automatically to internal cues that drive unhealthy behavioral patterns, ACT proposes a treatment that aims to promote a personal value-driven life, distress tolerance training, and conscious decision-making based on mindfulness practice [27]. Due to the relative novelty of this approach, only a few studies have been conducted to examine the efficacy of ACT for enhancing the psychological and physical well-being of people with overweight or obesity, and even fewer have tested the effects of these interventions over time. However, existing evidence supports the ability of ACT and mindfulness to address obesity-related physical and psychological distress despite a lack of studies with longer follow-ups [27,28,35,36].

The Mind&Life study aims to add further data to the existing literature that could help to inform the development of an urgently needed effective treatment that promotes the long-term psychological and physical well-being of people with overweight or obesity. To the best of our knowledge, this study will also be the first to test the effectiveness of ACT in conjunction with mindfulness in targeting weight-related issues in the Spanish context, while presenting a treatment that also places special emphasis on psychological well-being, because of its consonance with the VHL view. Behaviors based on personal values will be promoted, which will consequently bring about lifestyle changes that will enhance overall physical well-being. This will permit us to analyze whether a departure from a weight-normative model will, in some way, enhance treatment effectiveness as previously observed [46,72]. Additionally, this trial will also produce results that are generalizable for those seeking treatment, since participants will be attending primary care units for a weight management intervention. Moreover, if the present intervention is found to be effective, then we expect that it will be implemented by the Health Service of the Basque Country to treat patients dealing with excess weight-related problems.

Further, it is expected that the various process outcomes measured throughout this study will help to clarify the mechanisms of change or “active ingredients” of third-wave behavioral therapies, as Lawlor et al. suggested in a recent review [25]. We also plan to explore the mediating and moderating role of internalized weight stigma and emotional eating in treatment outcomes, given the importance of these psychological variables in obesity-related difficulties [7,31,33,73].

## 4. Conclusions

Overall, the aim of the current work is to provide data that could indicate the long-term effectiveness of ACT- and mindfulness-based treatments following a VHL approach for individuals with overweight or obesity. This work also aims to identify the specific processes that underpin any long-term behavioral changes observed while allowing us to ascertain whether a shift away from the weight-normative approach could be a viable option for promoting the overall well-being of these individuals.

## Figures and Tables

**Table 1:** TAU components.

Session Number	Content
1	Eating and physical activity-related instructions and recommendations
2	Food and physical activity pyramid and Harvard plate
3	Seasonally adapted weekly menu
4	Nutritional labelling
5	Recommendations for maintaining healthy habits

**Table 2:** Mind&Life intervention components.

Sn ^1^	Content and Processes	Key Metaphors and Exercises	Mindfulness Exercises
1	Creative hopelessness, values	Man in the hole metaphor	Mindfulness introduction
2	Control as the problem, willingness as the alternative, values	What are the numbers exercise, fall in love metaphor, polygraph metaphor, tug-of-war with a monster metaphor, butterfly metaphor, my shrinking life space exercise, living in the cottage or in the new house metaphor	Mindful eating
3	Distress tolerance, willingness, and acceptance, basic emotions, pain vs. suffering, values	Eyes on exercise, two scales metaphor, attending your own funeral metaphor	Contacting the present
4	The functioning of the mind I, observing thoughts, cognitive defusion	Take your mind for a walk exercise	Mindful eating, leaves on a stream
5	The functioning of the mind II, have a thought or buy it, cognitive defusion	Passengers on the bus metaphor	Bodily sensations
6	Values, committed action, distress tolerance	The values-focused vs. the goals-focused life metaphor, values in the trash exercise, urge surfing exercise	Acceptance mindfulness
7	The observing self, self as context	Paintings in the museum exercise, the label parade exercise, the sky and the weather metaphor	Observing-self
8	The observing self, self as context, self-compassion	Timeline exercise, chessboard metaphor, identification labels exercise	Mindful eating, Tonglen mindfulness
9	Acceptance, habit building, psychological flexibility and rigidity, defusion strategies	Change what you can and accept what you cannot exercise	Stay in the present moment, five senses
10	Review I, values	Physicalizing exercise, foreseeable and flexible answers exercise, milestone metaphor	Mountain mindfulness
11	Review II, social support management	Swamp metaphor, holding books exercise	Thought mindfulness
12	Mindless eating and mindful eating enhancement, defusion review	Mirror exercise, physicalizing exercise, unwelcome party guest metaphor	Emotions-centered mindfulness
13	Emotional eating, obstacles to living actively	Obstacles and strategies to live actively	Mindful eating
14	Lapse vs. relapse, live with courage, committed action	Epitaph metaphor	Leaves on a stream
15	Relapses, personal action plan, committed action	Path up the mountain metaphor	Mindful walking

^1^ Sn = session number.
